# Finite Element Study of Bio-Convective Stefan Blowing Ag-MgO/Water Hybrid Nanofluid Induced by Stretching Cylinder Utilizing Non-Fourier and Non-Fick’s Laws

**DOI:** 10.3390/nano11071735

**Published:** 2021-06-30

**Authors:** Puneet Rana, Vinita Makkar, Gaurav Gupta

**Affiliations:** 1 School of Mathematical Sciences, College of Science and Technology, Wenzhou-Kean University, Wenzhou 325060, China; prana@kean.edu or; 2 Department of Basic and Applied Sciences, School of Engineering and Sciences, GD Goenka University, Gurgaon 122103, India; vini252011makkar@gmail.com

**Keywords:** hybrid nanofluid, cylinder, stefan blowing, FEM, non-fourier, bioconvection

## Abstract

In the present framework, an analysis on nanofluid magneto-transport phenomena over an extending cylinder influenced by gyrotactic behavior of algal suspension, is made using the Cattaneo–Christov heat flux (non-Fourier) and mass flux (non-Fick’s) concept in modified Buongiorno’s model. Two dimensional incompressible MHD hybrid nanofluid which comprises chemically reactive hybrid nanomaterials (Ag-MgO NPs) and Stefan blowing effect along with multiple slips is considered. The experimental correlations with their dependency on initial nanoparticle volume fraction are used for viscosity and thermal conductivity of nanofluids. Similarity transformation is used to convert the governing PDE’s into non-linear ODE’s along with boundary conditions, which are solved using the Galerkin Finite Element Method (GFEM). The mesh independent test with different boundary layer thickness ($\xi_{\infty }$) has been conducted by taking both linear and quadratic shape functions to achieve a optimal desired value. The results are calculated for a realistic range of physical parameters. The validation of FEM results shows an excellent correlation with MATLAB *bvp5c* subroutine. The warmth exhibitions are assessed through modified version of Buongiorno’s model which effectively reflects the significant highlights of Stefan blowing, slip, curvature, free stream, thermophoresis, Brownian motion and bio-convection parameters. The present study in cylindrical domain is relevant to novel microbial fuel cell technologies utilizing hybrid nanoparticles and concept of Stefan blowing with bioconvection phenomena.

## 1. Introduction

A rapid progress has urged researchers’ attention over flow regime towards stretched cylinder. Fluid flow by a directly or non-straightly extending chamber assumes a critical part and have huge applications in assembling and creation measures including metal turning, creation of glass filaments, elastic sheet formation, wire drawing, expulsion of polymer sheets, petrol businesses, polymer preparing and so forth. The progression of quiet liquid over a moving surface was first broke down by Sakiadis [1]. Since most recent 20 years, the investigation of nanofluid has asked the scientist’s consideration because of their warmth transportation rate. Nanofluid comes into existence when we add a little amount of nano-sized particles to the base liquids. The term nanofluid was first instituted by Choi and Eastman [2]. In 2006, Buongiorno [3] explained two-component nanofluid modelling based on seven slip mechanisms which further utilized for heat transport investigation in nanofluid uniform layer by Kuznetsov and Nield [4]. Dhanai et al. [5] studied the variable slip effects over an inclined cylinder in presence of mixed convection. Swapna et al. [6] studied radiative effect over permeable stretching cylinder by using FE technique. Rana et al. [7] studied the energy dissipating effect of Viscoelastic Nanofluid Flow by using hp-FEM technique. Recently, Vinita and Poply [8] inspected influence of free stream velocity over stretching cylinder in MHD nanofluids. Goyal et al. [9] examined the GFEM examination in MHD nanofluid flow towards extending sheet and the FEM approach was implemented to solve system. Vinita et al. [10] explained the effect of variable slip flows in addition to thermal radiation in MHD nanofluids induced by non linear stretched surface. Vinita et al. [11] examined radiation effect on MHD free stream velocity nanofluid flow induced by stretchable cylinder in presence of chemically reactive species by applying RKF technique using ODE45 solver in MATLAB. Khan et al. [12] studied the applications of bio-convection nanofluid flow in presence of activation energy. The influence of Fick’s and Fourier laws on MHD dusty Casson nanofluid in presence of heat source parameter was investigated by Ramzan et al. [13].

In different related conditions of the heat move measure, probably the best guideline is exemplary Fourier heat law which is utilized in traditional material science [14]. One of its chief drawbacks of the constituting condition of heat is that particular aggravation and at the same time, the possibility of determinism are tested over the whole entire medium. Numerous specialists have clarified the conduct of pseudo-plastic liquids by recommending various models, especially Carreau, Williamson, power law, Upper Convective Maxwell (UCM), Cattaneo–Christov (Non-Fourier) Model, and so on. The Cattaneo’s thermal unwinding is the modifed type of Fourier’s thermal conduction law [15]. For Oldroyd’s super convection model, Christov [16] extended Cattaneo’s law [15] to achieve the invariance of structural elements by adding rest periods. Along these lines, another model called Cattaneo–Christov was introduced based on heat flux. Recently, Kumar et al. [17] examined the CNT’s based flow along with thermal convective conditions and Cattaneo–Christov (non-Fourier) model. Abid et al. [18] studied the effect of two distinctive nanoparticles Cu and CuO in two diverse part of the way ionized magneto-fluid over a straightly extending surface. Recently, the flow of the boundary layer and the heat transfer model with Cattaneo–Christov have been extensively investigated in many publications (see [19,20,21,22,23]).

Stagnation point nanofluid flow with Stefan blowing in presence of chemical reaction and heat radiation has been studied by Rana et al. [24] by following Homotopy analysis. Additionally, Rana et al. [25] analyzed the entropy generation impact along with Stefan blowing and slip flow induced by horizontal surface using Lie analysis approach and found that higher Reynolds number rises entropy generation number. Gowda et al. [26] investigated the Stefan blowing impact with magnetic dipole over stretching surface in ferromagnetic nanofluids by following RKF45 technique and showed that heat transportation of fluid is enhanced with Stefan blowing convective conditions. Impact of Stefan blowing over rotating disc in Maxwell nanofluids has been investigated by Mabood et al. [27] and found that higher thermal relaxation parameter falls temperature field. Recently, Gowda et al. [28] presented the Stefan effect with Cattaneo–Christov model in Sutterby nanofluids over rotating disc by extending Buongiorno’s model in their study. Additionally, Madhukesh et al. [29] used non-Fourier heat flux model over curved stretching surface to investigate the impact of AA7072-AA7075/water-based hybrid nanofluid along with Newtonian heating when temperature at the wall is constant. In 2021, two dimensional laminar flow of non-Newtonian Marangoni nanofluids in presence of activation energy along with chemical reactions has been presented by Gowda et al. [30] and they showed that heat transfer rate declines with larger Marangoni number. Very recently, Yusuf et al. [31] studied the bio-convective entropy generation effect induced by inclined plate in presence of Williamson nanofluid.

To achieve stability of hybrid nanofluids, the proper selection of NPs plays a significant role for base fluids. Various examinations have been directed to test the qualities of crossover nanofluids. Dominant parts of the analysts have announced empowering results. Hybrid nanofluids have been found to have streamlined qualities, demonstrating them to be appropriate for the heavenly bodies that require great warm, optical, and rheological attributes of working liquid. Flow caused by $Al_{2}O_{3}$ water-based nanofluid by shrinking cylinder in the presence of slip conditions was examined by Rana et al. [32] by following Buongiorno’s model. Esfe et al. [33] explained that hybrid nanofluid have better influence on thermal conductivity than single particle nanofluids. Following segment sums up significant properties such as optical, warm, rheological, and morphological properties of mixture nanofluids according to revealed in the latest examination articles presented by Shah and Ali [34]. MHD hybrid nanofluid flow in annulus among concentric cylinders was studied by Rana [35]. A numerical investigation was done by Aminian et al. [36] to study the impact of Magnetic by extending cylinder in hybrid nanofluids. Gul et al. [37] showed the impact of magnetic dipole over stretching surface in hybrid nanofluid flow. Reddy et al. [38] explained the effect of CCHFM over hybrid dusty nanofluids. Khashi’ie et al. [39] examined hybrid nanofluid flow with prescribed surface heat flux Past a shrinking cylinder. Tassaddiq [40] examined the influence of CCHFM on MHD hybrid nanofluid flow in presence of viscous and Ohmic dissipation and found that macro velocity falls for higher values of Hartmann number and micropolar factor. In recent years, hybrid nanofluid flow of the boundary layer and the heat transfer model with Cattaneo–Christov in different circumstances have been extensively investigated in many publications [41,42,43].

An important point of current research is the outstanding investigation of hybrid nanofluid flow through CCHFM along with Stefan blowing, mass flux, chemical reaction and gyrotactic micro-organism over a stretched cylinder. The ruling PDEs are converted as ODE’s to obtain numerical solution using Finite Element technique with different shape functions. The results are also compared with MATLAB finite difference *bvp5c* function. A catalog who works well for the physical steps to solve this model are presented and correlation between flow and temperatures represented by tables and graphs. Finally, the physical quantities of interest are presented for various controlling parameters in the form of contours.

## 2. Nano-Materials and Modeling

**Figure 1:**
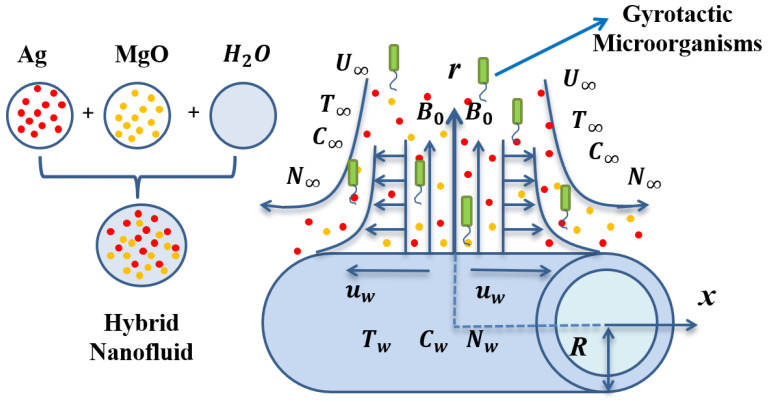
Physical model with co-ordinate system.

Two dimensional incompressible MHD hybrid nanofluid (Ag-MgO/water) flow with gyrotactic micro-organisms in presence of chemically reactive species towards an extended surface with free stream velocity ($U_{\infty }$) has been taken into account (See Figure 1). The flow field is impacted by the gradient of nanoparticle volume fraction at the wall (Stefan blowing phenomena) with low magnetic Reynolds number. The cylinder is stretched with stretching velocity $u_{w}=ax/L$ in direction of *x*-axis whereas magnetic force has been employed in the direction of radial axis. Additionally, free stream motile organisms, nanoparticle volume fraction and temperature are expressed by $N_{\infty }$, $C_{\infty }$ and $T_{\infty }$ respectively. The governing equations for nanofluid are depicted as:

(1) $$\frac{\partial }{\partial x}\left(ru\right)+\frac{\partial }{\partial r}\left(rv\right)=0$$

(2) $$u\frac{\partial u}{\partial x}+v\frac{\partial u}{\partial r}=\frac{\mu_{hnf}}{\rho_{hnf}}\left(\frac{\partial^{2}u}{\partial r^{2}}+\frac{1}{r}\frac{\partial u}{\partial r}\right)-\frac{\sigma_{hnf}{B_{0}}^{2}}{\rho_{hnf}}\left(u-U_{\infty }\right)+U_{\infty }\frac{\partial U_{\infty }}{\partial x}$$

(3) $$\begin{array}{c} u\frac{\partial T}{\partial x}+v\frac{\partial T}{\partial r}+\lambda_{E}\left[v\frac{\partial v}{\partial r}\frac{\partial T}{\partial r}+v\frac{\partial u}{\partial r}\frac{\partial T}{\partial x}+u\frac{\partial v}{\partial x}\frac{\partial T}{\partial r}+u\frac{\partial u}{\partial x}\frac{\partial T}{\partial x}+2uv\frac{\partial^{2}T}{\partial x\partial r}+v^{2}\frac{\partial^{2}T}{\partial r^{2}}+u^{2}\frac{\partial^{2}T}{\partial x^{2}}\right]\\ =\frac{k_{hnf}}{{\left(\rho c\right)}_{hnf}}\left(\frac{\partial^{2}T}{\partial r^{2}}+\frac{1}{r}\frac{\partial T}{\partial r}\right)+\frac{{\left(\rho c\right)}_{p}}{{\left(\rho c\right)}_{hnf}}\left[D_{B}\frac{\partial C}{\partial r}\frac{\partial T}{\partial r}+\frac{D_{T}}{T_{\infty }}{\left(\frac{\partial T}{\partial r}\right)}^{2}\right] \end{array}$$

(4) $$\begin{array}{c} u\frac{\partial C}{\partial x}+v\frac{\partial C}{\partial r}+\lambda_{C}\left[v\frac{\partial v}{\partial r}\frac{\partial C}{\partial r}+v\frac{\partial u}{\partial r}\frac{\partial C}{\partial x}+u\frac{\partial v}{\partial x}\frac{\partial C}{\partial r}+u\frac{\partial u}{\partial x}\frac{\partial C}{\partial x}+2uv\frac{\partial^{2}C}{\partial x\partial r}+v^{2}\frac{\partial^{2}C}{\partial r^{2}}+u^{2}\frac{\partial^{2}C}{\partial x^{2}}\right]\\ =D_{B}\left(\frac{\partial^{2}C}{\partial r^{2}}+\frac{1}{r}\frac{\partial C}{\partial r}\right)+\frac{D_{T}}{T_{\infty }}\left(\frac{\partial^{2}T}{\partial r^{2}}+\frac{1}{r}\frac{\partial T}{\partial r}\right)-K_{r}\left(C-C_{\infty }\right) \end{array}$$

(5) $$u\frac{\partial N}{\partial x}+v\frac{\partial N}{\partial r}+\frac{dW_{c}}{C_{w}-C_{\infty }}\left[\frac{\partial }{\partial r}\left(N\frac{\partial C}{\partial r}\right)\right]=D_{N}\left(\frac{\partial^{2}N}{\partial r^{2}}+\frac{1}{r}\frac{\partial N}{\partial r}\right)-K_{r}\left(N-N_{\infty }\right)$$

Here *v* and *u* represents radial velocity and horizontal velocity. Additionally, $T_{\infty }$ stands for ambient temperature, $D_{B}$ stands for Brownian diffusion coefficient, $\lambda_{E}$ denoted the thermal relaxation parameter, *T* stands for temperature, $\lambda_{C}$ stands for nanoparticle volume fraction relaxation parameter, $D_{T}$ stands for thermophoresis diffusion coefficient, $K_{r}$ is the chemical reaction parameter, *C* stands for nanoparticle volume fraction, $B_{0}$ stands for magnetic field intensity, $\sigma_{hnf}$ stands for electrical conductivity, $\nu_{hnf}$ stands for kinematic viscosity and $U_{\infty }$ stands for free stream velocity. In Equation (5), *d* is constant, $W_{c}$ is maximum swimming speed of micro-organisms in hybrid-nanofluid and $D_{N}$ is diffusivity of micro-organisms.

Associated boundary conditions:

(6) $$\begin{array}{c} u=u_{w}+N_{1}\frac{\partial u}{\partial r},\;v=-\frac{D_{B}}{1-C_{w}}\left(\frac{\partial C}{\partial r}\right),\;T=T_{w}+N_{2}\left(\frac{\partial T}{\partial r}\right),\;C=C_{w}+N_{3}\left(\frac{\partial C}{\partial r}\right),\\ \;N=N_{w}+N_{4}\left(\frac{\partial N}{\partial r}\right)\;at\;r=R,\\ \mathrm{and}\;u\to U_{\infty }=\frac{bx}{L},\;T\to T_{\infty },\;C\to C_{\infty },\;N\to N_{\infty }\;as\;r\to \infty \end{array}$$

The thermophysical properties of basefluid and nanoparticles (Ag and MgO) are shown in Table 1. The correlations for electrical conductivity, heat capacitance, density, dynamic viscosity and thermal conductivity of Ag–MgO/water hybrid nanofluid with the particle diameter of 40 nm (MgO) and 25 nm (Ag) and nanoparticle volume fraction (50% Ag and 50% MgO by volume), are specified as [44,45,46]:

(7) $$\sigma_{r}=\frac{\sigma_{hnf}}{\sigma_{f}}=\left[\frac{1+3\left(\frac{\sigma }{\sigma_{f}}-1\right)\left(\phi_{Ag}+\phi_{MgO}\right)}{\left(\frac{\sigma }{\sigma_{f}}+2\right)-\left(\frac{\sigma }{\sigma_{f}}-1\right)\left(\phi_{Ag}+\phi_{MgO}\right)}\right]$$

(8) $${\left(\rho c\right)}_{r}=\frac{{\left(\rho c\right)}_{hnf}}{{\left(\rho c\right)}_{f}}=\left(1-\phi_{Ag}-\phi_{MgO}\right)+\phi_{Ag}\frac{{\left(\rho c\right)}_{Ag}}{{\left(\rho c\right)}_{f}}+\phi_{MgO}\frac{{\left(\rho c\right)}_{MgO}}{{\left(\rho c\right)}_{f}}$$

(9) $$\rho_{r}=\frac{\rho_{hnf}}{\rho_{f}}=\left(1-\phi_{Ag}-\phi_{MgO}\right)+\phi_{Ag}\frac{\rho_{Ag}}{\rho_{f}}+\phi_{MgO}\frac{\rho_{MgO}}{\rho_{f}}$$

(10) $$\mu_{r}=\frac{\mu_{hnf}}{\mu_{f}}=\left[1+32.795\phi_{1}-7214{\phi_{1}}^{2}+714600{\phi_{1}}^{3}-0.1941\times 10^{8}{\phi_{1}}^{4}\right];\;0\leq \phi_{1}\leq 0.02$$

(11) $$\begin{array}{c} k_{r}=\frac{k_{hnf}}{k_{f}}=\left[\frac{0.1747\times 10^{5}+\phi_{1}}{0.1747\times 10^{5}-0.1498\times 10^{6}\phi_{1}+0.1117\times 10^{7}{\phi_{1}}^{2}+0.1997\times 10^{8}{\phi_{1}}^{3}}\right];\\ 0\leq \phi_{1}\leq 0.03 \end{array}$$

Additionally, the similarity variables are specified by following:

(12) $$\begin{array}{c} \xi =\frac{r^{2}-R^{2}}{2R}{\left(\frac{u_{w}\rho_{f}}{x\mu_{f}}\right)}^{\frac{1}{2}},\;\;\;\;\;\psi ={\left(\frac{u_{w}x\mu_{f}}{\rho_{f}}\right)}^{\frac{1}{2}}Rf\left(\xi \right),\;\;\;\;\;\theta \left(\xi \right)=\frac{T-T_{\infty }}{T_{w}-T_{\infty }}\;\;\;\;\;\Phi \left(\xi \right)=\frac{C-C_{\infty }}{C_{w}-C_{\infty }}\\ \mathrm{and}\;\;\;\;\;\chi \left(\xi \right)=\frac{N-N_{\infty }}{N_{w}-N_{\infty }} \end{array}$$

Inserting (12) into Equations (2)–(5), we have a system of the following differential equations:

(13) $$\mu_{r}\left[\left(1+2\xi \gamma \right)f^{{}^{\prime \prime \prime }}+2\gamma f^{{}^{\prime \prime }}\right]+\rho_{r}\left[ff^{{}^{\prime \prime }}-{f^{{}^{\prime }}}^{2}+\epsilon^{2}\right]-\sigma_{r}M^{2}\left(f^{{}^{\prime }}-\epsilon \right)=0$$

(14) $$\begin{array}{c} k_{r}\left[\left(1+2\xi \gamma \right)\theta^{{}^{\prime \prime }}+2\gamma \theta^{{}^{\prime }}\right]+PrNb\left(1+2\xi \gamma \right)\theta^{{}^{\prime }}\Phi^{{}^{\prime }}+PrNt\left(1+2\xi \gamma \right){\theta^{{}^{\prime }}}^{2}\\ +{\left(\rho c\right)}_{r}Pr\left[f\theta^{{}^{\prime }}-\alpha_{t}\left(f^{2}\theta^{{}^{\prime \prime }}+ff^{{}^{\prime }}\theta^{{}^{\prime }}\right)\right]=0 \end{array}$$

(15) $$\begin{array}{c} \left(1+2\xi \gamma \right)\Phi^{{}^{\prime \prime }}+Lef\Phi^{{}^{\prime }}+2\gamma \Phi^{{}^{\prime }}-Le\;\alpha_{c}\left(f^{2}\Phi^{{}^{\prime \prime }}+ff^{{}^{\prime }}\Phi^{{}^{\prime }}\right)\\ +\left(1+2\xi \gamma \right)\frac{Nt}{Nb}\theta^{{}^{\prime \prime }}+2\gamma \frac{Nt}{Nb}\theta^{{}^{\prime }}-LeC_{r}\Phi =0 \end{array}$$

(16) $$\begin{array}{c} \left(1+2\xi \gamma \right)\chi^{{}^{\prime \prime }}+Lbf\chi^{{}^{\prime }}+2\gamma \chi^{{}^{\prime }}-\\ Pe\left[\left(1+2\xi \gamma \right)\chi \Phi^{{}^{\prime \prime }}+\gamma \chi \Phi^{{}^{\prime }}+\Omega_{1}\gamma \Phi^{{}^{\prime }}\right.\left.+\Omega_{1}\left(1+2\xi \gamma \right)\Phi^{{}^{\prime \prime }}+\left(1+2\xi \gamma \right)\chi^{{}^{\prime }}\Phi^{{}^{\prime }}\right]-LbC_{r}\chi =0 \end{array}$$

with

(17) $$\begin{array}{c} f^{\prime }\left(0\right)=1+\delta_{1}f^{\prime \prime }\left(0\right),\;f\left(0\right)=\frac{Sb}{Le\;Pr}\Phi^{\prime }\left(0\right),\;\theta \left(0\right)=1+\delta_{2}\theta^{\prime }\left(0\right),\;\Phi \left(0\right)=1+\delta_{3}\Phi^{\prime }\left(0\right),\;\\ \chi \left(0\right)=1+\delta_{4}\chi^{\prime }\left(0\right)\\ \mathrm{and}\;f^{\prime }\left(\xi \right)\to \epsilon ,\;\theta \left(\xi \right)\to 0,\;\Phi \left(\xi \right)\to 0,\;\chi \left(\xi \right)\to 0\;as\;\xi \to \infty \end{array}$$

where crucial fluid dimensionless parameters are specified as:

(18) $$\begin{array}{c} \alpha_{t}=\frac{a\;\lambda_{E}}{L},\;\alpha_{c}=\frac{a\;\lambda_{C}}{L},\;C_{r}=\frac{K_{r}L}{a},\;Le=\frac{\mu_{f}}{\rho_{f}D_{B}},\;Nb=\frac{{\left(\rho c\right)}_{p}D_{B}\left(C_{w}-C_{\infty }\right)}{{\left(\rho c\right)}_{f}\nu_{f}},\;\;\;\epsilon =\frac{b}{a},\;\\ Pr=\frac{\mu_{f}}{\rho_{f}\alpha_{f}},\;\gamma =\frac{1}{R}\sqrt{\frac{\mu_{f}L}{\rho_{f}a}},\;Nt=\frac{{\left(\rho c\right)}_{p}D_{T}\left(T_{w}-T_{\infty }\right)}{{\left(\rho c\right)}_{f}T_{\infty }\nu_{f}},\;M^{2}=\frac{\sigma_{f}{B_{0}}^{2}L}{\rho_{f}a},\;\;\;Sb=\frac{C_{w}-C_{\infty }}{1-C_{w}},\;\;\;\\ Pe=\frac{dW_{c}}{D_{N}},\;\;\Omega_{1}=\frac{N_{\infty }}{N_{w}-N_{\infty }},\;\;Lb=\frac{\mu_{f}}{\rho_{f}D_{N}},\;\delta_{i}=N_{i}\sqrt{\frac{c\rho_{f}}{L\mu_{f}}}\left(i=1,\ldots ,4\right),\;\alpha_{f}=\frac{k_{f}}{{\left(\rho c\right)}_{f}}. \end{array}$$

The skin friction coefficient $Cf_{r}$, local Nusselt number $Nu_{r}$, local Sherwood number $Sh_{r}$ and local motile micro-organism number $M_{r}$ are defined as

(19) $$Cf=\frac{\tau_{w}}{\rho U_{\infty }^{2}},Nu=\frac{xq_{w}}{k(T_{f}-T_{\infty })},Sh=\frac{xq_{m}}{D_{B}\left(C_{w}-C_{\infty }\right)},Mo=\frac{xq_{n}}{D_{N}\left(N_{w}-N_{\infty }\right)},$$

where the wall shear stress $\tau_{w}$, the local heat flux $q_{w}$, the local mass flux $q_{m}$ and local micro-organism mass flux $q_{n}$ as follows

(20) $$\tau_{w}=\mu_{hnf}{\left(\frac{\partial u}{\partial r}\right)}_{r=R},q_{w}=-k_{hnf}{\left(\frac{\partial T}{\partial r}\right)}_{r=R},q_{m}=-D_{B}{\left(\frac{\partial C}{\partial r}\right)}_{r=R},q_{n}=-D_{N}{\left(\frac{\partial N}{\partial r}\right)}_{r=R},$$

where $\mu_{hnf}$ is the dynamic viscosity of hybrid nanofluid. Using the variables (12), the skin friction coefficient, local Nusselt number, local Sherwood number and local mobile micro-organism density number are given below:

(21) $$\begin{array}{c} Cf_{r}=CfRe_{x}^{1/2}=\mu_{r}f^{\prime \prime }\left(0\right),\;\;Nu_{r}=NuRe_{x}^{-1/2}=-k_{r}\theta^{\prime }\left(0\right),Sh_{r}=ShRe_{x}^{-1/2}=-\Phi^{\prime }\left(0\right),\\ M_{r}=MoRe_{x}^{-1/2}=-\chi^{\prime }\left(0\right), \end{array}$$

where $Re_{x}=u_{w}x/\nu_{f}$ is the local Reynold number.

## 3. Numerical Method

**Figure 2:**
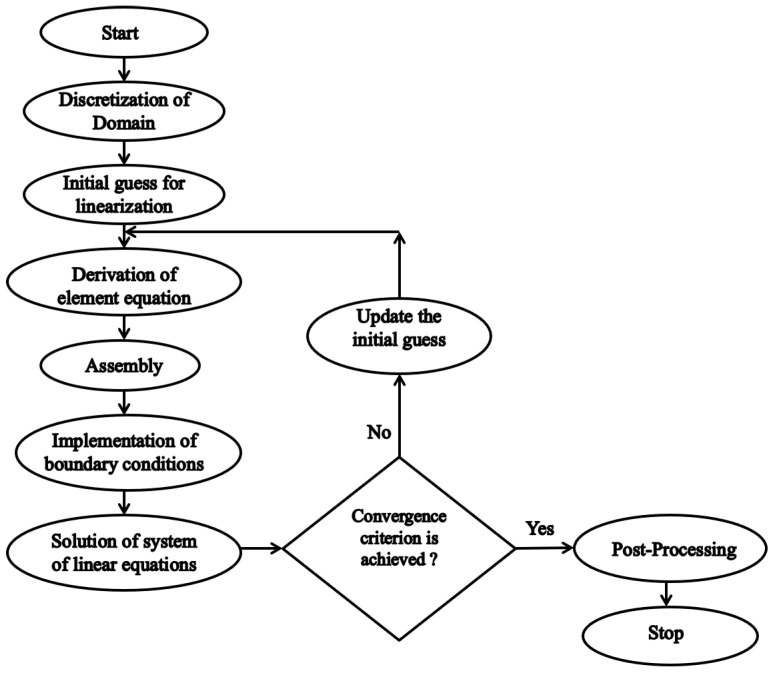
FEM Working Flow Chart

The Galerkin finite element approach (GFEM) is one of the well-known numerical strategies to discover approximate solutions of ODE in addition to PDE system that includes complex boundary situations and/or complicated geometry. The working of finite element method are shown in Figure 2. To solve this system of non-linear differential Equations (13)–(16) following (Swapna et al. [6], Rana et al. [7], Goyal et al. [9]), the weak formulation procedure has been adopted and $\Sigma_{e}$ represents the typical linear/quadratic element that is created by weighted residual formulation having element coordinates $\left(\xi_{e},\xi_{e+1}\right)$, is given by

(22) $$\int_{\Sigma_{e}}w_{1}\left\{f^{\prime }-h\right\}d\xi =0$$

(23) $$\int_{\Sigma_{e}}w_{2}\left\{\frac{d}{d\xi }\left[\mu_{r}\left(1+2\xi \gamma \right)h^{\prime }\right]+\rho_{r}\left[fh-h^{2}+\epsilon^{2}\right]-\sigma_{r}M\left(h-\epsilon \right)\right\}d\xi =0$$

(24) $$\int_{\Sigma_{e}}w_{3}\left\{\begin{array}{c} \frac{d}{d\xi }\left[k_{r}\left(1+2\xi \gamma \right)\theta^{\prime }\right]+\mathrm{Pr}Nb\left(1+2\xi \gamma \right)\theta^{\prime }\Phi^{\prime }+\mathrm{Pr}Nt\left(1+2\xi \gamma \right){\theta^{\prime }}^{2}\\ +{\left(\rho c\right)}_{r}\mathrm{Pr}\left[f\theta^{\prime }-\alpha_{t}f\frac{d}{d\xi }\left[f^{\prime }\theta^{\prime }\right]\right] \end{array}\right\}d\xi =0$$

(25) $$\begin{array}{c} \int_{\Sigma_{e}}w_{4}\left\{\frac{d}{d\xi }\left[\left(1+2\xi \gamma \right)\Phi^{\prime }\right]+Lef\Phi^{\prime }+\frac{Nt}{Nb}\frac{d}{d\xi }\left[\left(1+2\xi \gamma \right)\theta^{\prime }\right]-Le\left[\alpha_{c}f\frac{d}{d\xi }\left[f^{\prime }\Phi^{\prime }\right]+Cr\Phi \right]\right\}d\xi =0 \end{array}$$

(26) $$\int_{\Sigma_{e}}w_{5}\left\{\begin{array}{c} \frac{d}{d\xi }\left[\left(1+2\xi \gamma \right)\chi^{\prime }\right]+Lbf\chi^{\prime }-Pe\left[\frac{d}{d\xi }\left[\left(1+2\xi \gamma \right)\left(\chi +\Omega_{1}\right)\Phi^{\prime }\right]-\gamma \left(\chi +\Omega_{1}\right)\Phi^{\prime }\right]\\ -LbCr\chi \end{array}\right\}d\xi =0$$

Here, we choose $w_{1},w_{2},w_{3}$, $w_{4}$ and $w_{5}$ as the test functions which are variants of *f*, *h*, θ, Φ and χ respectively. Additionally, the dependent variable Θ having form Θ = $\sum_{i=1}^{m}\Theta_{i}S_{i}$ is assumed where Θ stands for either *f*, *h*, θ, Φ and χ with $w_{1}=w_{2}=w_{3}=w_{4}=w_{5}=S_{j}$, (j=1,…,5). Using both linear and quadratic shape functions, finite element representation is now formulated as:

$$\begin{array}{l} \left[\begin{array}{ccccc} \left[M^{11}\right] & \left[M^{12}\right] & \left[M^{13}\right] & \left[M^{14}\right] & \left[M^{15}\right]\\ \left[M^{21}\right] & \left[M^{22}\right] & \left[M^{23}\right] & \left[M^{24}\right] & \left[M^{25}\right]\\ \left[M^{31}\right] & \left[M^{32}\right] & \left[M^{33}\right] & \left[M^{34}\right] & \left[M^{35}\right]\\ \left[M^{41}\right] & \left[M^{42}\right] & \left[M^{43}\right] & \left[M^{44}\right] & \left[M^{45}\right]\\ \left[M^{51}\right] & \left[M^{52}\right] & \left[M^{53}\right] & \left[M^{54}\right] & \left[M^{55}\right] \end{array}\right]\left[\begin{array}{c} f\\ h\\ \theta \\ \Phi \\ \chi \end{array}\right]=\left[\begin{array}{c} \left\{t^{1}\right\}\\ \left\{t^{2}\right\}\\ \left\{t^{3}\right\}\\ \left\{t^{4}\right\}\\ \left\{t^{5}\right\} \end{array}\right] \end{array}$$

where $\left[M^{mn}\right],\left\{t^{m}\right\}$, (m,n)=1,…,5 are established as:

(27) $$M_{ij}^{11}=\int_{\Sigma_{e}}S_{i}{S^{\prime }}_{j}d\xi ,\;\;M_{ij}^{12}=-\int_{\Sigma_{e}}S_{i}S_{j}d\xi ,M_{ij}^{13}=M_{ij}^{14}=M_{ij}^{15}=0,$$

(28) $$\begin{array}{cc} \; & M_{ij}^{21}=0,M_{ij}^{22}=-\mu_{r}\int_{\Sigma_{e}}\left(1+2\gamma \xi \right){S^{\prime }}_{i}{S^{\prime }}_{j}d\xi +\rho_{r}\int_{\Sigma_{e}}\bar{f}S_{i}{S^{\prime }}_{j}d\xi -\rho_{r}\int_{\Sigma_{e}}\bar{h}S_{i}S_{j}d\xi -\sigma_{r}M\int_{\Sigma_{e}}S_{i}S_{j}d\xi ,\\ & M_{ij}^{23}=0,M_{ij}^{24}=0,M_{ij}^{25}=0, \end{array}$$

(29) $$\begin{array}{cc} \; & M_{ij}^{31}=M_{ij}^{32}=M_{ij}^{35}=0,M_{ij}^{33}=-k_{r}\int_{\Sigma_{e}}\left(1+2\gamma \xi \right){S^{\prime }}_{i}{S^{\prime }}_{j}d\xi +{\left(\rho c\right)}_{r}\mathrm{Pr}\int_{\Sigma_{e}}\bar{f}S_{i}{S^{\prime }}_{j}d\xi \\ \; & +\mathrm{Pr}Nt\int_{\Sigma_{e}}\left(1+2\gamma \xi \right)S_{i}\bar{\theta^{\prime }}S_{j}d\xi +{\left(\rho c\right)}_{r}\mathrm{Pr}\alpha_{t}\int_{\Sigma_{e}}\bar{f}\bar{h}S_{i}S_{j}d\xi +{\left(\rho c\right)}_{r}\mathrm{Pr}\alpha_{t}\int_{\Sigma_{e}}\bar{f^{2}}S_{i}{S^{\prime }}_{j}d\xi ,\\ & M_{ij}^{34}=\mathrm{Pr}Nb\int_{\Sigma_{e}}\left(1+2\gamma \xi \right)S_{i}\bar{\theta^{\prime }}S_{j}d\xi , \end{array}$$

(30) $$\begin{array}{cc} \; & M_{ij}^{41}=M_{ij}^{42}=M_{ij}^{45}=0,M_{ij}^{43}=-\frac{Nt}{Nb}\int_{\Sigma_{e}}\left(1+2\gamma \xi \right){S^{\prime }}_{i}{S^{\prime }}_{j}d\xi ,\\ & M_{ij}^{44}=-\int_{\Sigma_{e}}\left(1+2\gamma \xi \right){S^{\prime }}_{i}{S^{\prime }}_{j}d\xi +Le\int_{\Sigma_{e}}\bar{f}S_{i}{S^{\prime }}_{j}d\xi +Le\alpha_{c}\int_{\Sigma_{e}}\bar{f}\bar{h}S_{i}S_{j}d\xi +Le\alpha_{c}\int_{\Sigma_{e}}\bar{f^{2}}S_{i}{S^{\prime }}_{j}d\xi \\ \; & \;\;\;\;\;\;\;\;\;\;\;\;\;\;-LeCr\int_{\Sigma_{e}}S_{i}S_{j}d\xi , \end{array}$$

(31) $$\begin{array}{c} M_{ij}^{51}=M_{ij}^{52}=M_{ij}^{53}=0,M_{ij}^{54}=Pe\Omega_{1}\int_{\Sigma_{e}}\left(1+2\gamma \xi \right){S^{\prime }}_{i}{PS^{\prime }}_{j}d\xi +Pe\int_{\Sigma_{e}}\left(1+2\gamma \xi \right)\bar{\chi }S_{i}{PS^{\prime }}_{j}d\xi \\ +Pe\int_{\Sigma_{e}}\gamma \bar{\chi }S_{i}{PS^{\prime }}_{j}d\xi +Pe\int_{\Sigma_{e}}\gamma \Omega_{1}S_{i}{PS^{\prime }}_{j}d\xi ,\\ M_{ij}^{55}=-\int_{\Sigma_{e}}\left(1+2\gamma \xi \right){S^{\prime }}_{i}{PS^{\prime }}_{j}d\xi +Lb\int_{\Sigma_{e}}\bar{f}S_{i}{PS^{\prime }}_{j}d\xi -LbCr\int_{\Sigma_{e}}S_{i}S_{j}d\xi . \end{array}$$

(32) $$\begin{array}{c} t_{i}^{1}=0,t_{i}^{2}=-{\left({\left(1+2\xi \gamma \right)\mu_{r}S}_{i}\frac{dh}{d\xi }\right)}_{\xi_{e}}^{\xi_{e+1}}-\int_{\Sigma_{e}}(M\epsilon +\rho_{r}\epsilon^{2})S_{i}d\xi ,\\ t_{i}^{3}={\left(S_{i}\left[-\left(1+2\xi \gamma \right)k_{r}+{\left(\rho c\right)}_{r}Pr\alpha_{t}f^{2}\right]\frac{d\theta }{d\xi }\right)}_{\xi_{e}}^{\xi_{e+1}}, \end{array}$$

(33) $$\begin{array}{c} t_{i}^{4}={\left(S_{i}\left(\left[-\left(1+2\xi \gamma \right)k_{r}+{\left(\rho c\right)}_{r}Pr\alpha_{c}f^{2}\right]\frac{d\Phi }{d\xi }-\left(1+2\xi \gamma \right)\frac{Nt}{Nb}\frac{d\theta }{d\xi }\right)\right)}_{\xi_{e}}^{\xi_{e+1}},\\ t_{i}^{5}={\left(S_{i}\left(\left[-(1+2\xi \gamma )\right]\frac{d\chi }{d\xi }+\left(Pe+\Omega \right)\left(1+2\xi \gamma \right)\frac{d\Phi }{d\xi }\right)\right)}_{\xi_{e}}^{\xi_{e+1}}. \end{array}$$

(34) $$\bar{f}=\sum_{i=1}^{m}\bar{f_{i}}S_{i},\bar{h}=\sum_{i=1}^{m}\bar{h_{i}}S_{i},\;\;\;\bar{\theta^{\prime }}=\sum_{i=1}^{m}\bar{\theta_{i}}S_{i}^{\prime },\;\;\bar{\chi }=\sum_{i=1}^{m}\bar{\chi_{i}}S_{i}$$

where, m=2 corresponds to linear shape function and m=3 to quadratic shape function. Similarly, the right hand side column can be evaluated after weak formulation. In this physical configuration, whole domain is alienated into the equal length of both linear and quadratic elements which further solved after implementing the boundary conditions. The Gaussian quadrature method are used to solve the integration maintaining the accuracy of $0.5\times 10^{-8}$. The desired convergence has been achieved in current problem with optimal element size of 0.01 with boundary layer length of $\xi_{\infty }=10$ which can be observed from Table 2 and Table 3. The accuracy of the employed method (Finite Element Method) is also established by direct comparisons. We have compared the results obtained by FEM Results with those of a standard MATLAB built-in function *bvp5c* (Finite Difference Algorithm) as shown in Table 4. We have also noticed that MATLAB *bvp5c* provides convergence only for some set of controlling parameters. Thus, finite element method is advantageous for solving such a complex system.

## 4. Interpretation of Results

In the current investigation, nonlinear differential Conditions (13) to (16) with (17) are solved numerically using GFEM. Additionally, the impact of numerous fluid parameters, in particular dimensionless magnetic parameter *M*, velocity slip parameter $\delta_{1}$, curvature parameter γ, free stream velocity ϵ, initial volume fraction $\phi_{1}$, Brownian motion parameter Nb, Peclet number Pe, thermal slip parameter $\delta_{2}$, chemical reaction parameter $C_{r}$, volume fraction slip parameter $\delta_{3}$, motile concentration parameter $\delta_{4}$ and Stefan blowing Sb are addressed and clarified through plots and tables (Table 5 and Table 6). For numerical simulation, we have fixed the controlling parameters as M=0.1 (for magnetic field less than 0.1 Tesla), Nb=Nt=0.01 (<<1 for Ag/MgO hybrid nanoparticles), Pr=6.2 (water as base fluid), Le=Lb=10 (>>1, high for nanoparticles and algae micro-organisms), $C_{r}=1$ (>0, chemical reaction parameter), γ=0.1 (>0 for cylinder, =0 for plate model), ϵ=0.1 (stretching velocity is assumed to be higher than free-stream velocity), $\Omega_{1}=0.1$, Pe=1, $\delta_{1}=\delta_{2}=\delta_{3}=\delta_{4}=0.1$ (generally its value ≤1) and $\phi_{1}=0.01$ (nanoparticle initial volume fraction not more than 2%). The bioconvection parameters can be calculated for alga Chlamydomonas nivalis micro-organism using the data provided in Pedley [47] and Khurana et al. [48]. To sort out the computational non-linearity in the mathematical model, the finite element method (FEM) is executed to settle administering differential conditions, since it gives the flexibility to linearize along with polynomial approximation and shows a very good agreement of convergence in the present study.

### 4.1. Influence of Physical Parameters on Velocity and Temperature Profiles

**Figure 3:**
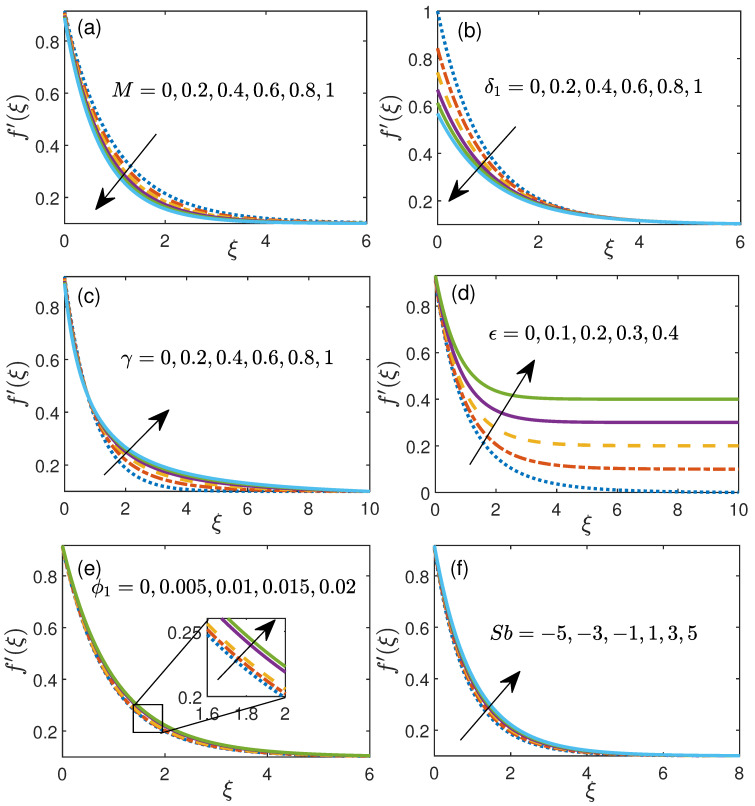
Velocity profile for (**a**) magnetic parameter *M*, (**b**) velocity slip parameter $\delta_{1}$, (**c**) curvature parameter γ, (**d**) free stream velocity ϵ, (**e**) initial volume fraction $\phi_{1}$ and (**f**) Stefan blowing Sb.

Figure 3 shows the velocity profile for magnetic parameter *M*, velocity slip parameter $\delta_{1}$, curvature parameter γ, free stream velocity ϵ, initial nanoparticle volume fraction $\phi_{1}$ and Stefan blowing Sb. Figure 3a portrays the influence of magnetic parameter *M* (0 to 1) over velocity distribution. Lorentz force is delivered in view of presence of an attractive field which opposes the free movement of electrically conducting basefluid (ionized) and it is the main reason behind the diminishing of velocity of nanofluid. Figure 3b addresses the velocity profile against slip velocity $\delta_{1}$ (0 to 1). Here, it is seen that nanofluid velocity diminishes with the enhancement in slip velocity $\delta_{1}$, may be due to flow velocity near the cylindrical surface is not same as speed of the extending cylinder. Moreover, it is obvious from the figure that all the charts decline particularly up to ξ=5 (approximately). Figure 3c address the profile of velocity for precise sections of curvature parameter γ (0 to 1). With the increment of γ, this diagram shows that velocity conveyance increments. Additionally, the cylindrical radius diminishes with expanding estimation of γ and subsequently the piece of chamber that is in contact to liquid is scaled down which brings about the decrease of nanoliquid obstruction and in this way the augmentation in profile of velocity is taken note. Figure 3d manifests impact of the free stream velocity ϵ against the velocity distribution for ϵ=0−0.4. For ϵ<1, it signifies the stretching sheet velocity ($u_{w}$) is assumed to be more than free-stream velocity ($U_{\infty }$). This plot describes that an enormous estimation of ϵ rises the velocity distribution and is approximately diminished at the surface of cylinder. Impact of initial volume fraction $\phi_{1}$ (0 to 0.02) over the velocity is shown by Figure 3e. This graph shows that velocity increases with a very slow speed with the increment in $\phi_{1}$. Additionally, the Figure 3f illustrates the velocity distribution against Stefan blowing parameter Sb (−5 to 5). A critical deviations in profile of velocity can be seen in this figure. With increase in the value of Sb, velocity increases as depicted in Figure 3f.

**Figure 4:**
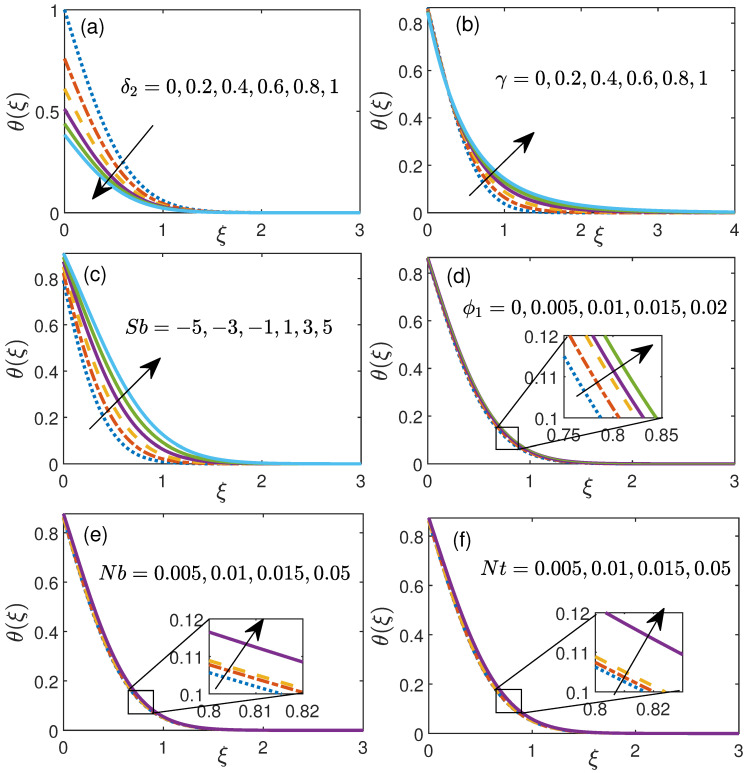
Temperature profile for (**a**) thermal slip parameter $\delta_{2}$, (**b**) curvature parameter γ, (**c**) Stefan blowing Sb, (**d**) initial volume fraction $\phi_{1}$, (**e**) Brownian motion Nb, (**f**) thermophoresis Nt.

Figure 4 illustrates the temperature profile for six crucial parameters. Figure 4a manifests the temperature distribution against thermal slip flow $\delta_{2}$ (0 to 1). This plot illustrates that for higher $\delta_{2}$, reduction in boundary layer thickness has been observed which consequently results in decrease of temperature and the outcome are highly apparent in the boundary layer 0≤ξ≤1.5 (approximately).

Figure 4b addresses the temperature profile for expanding curvature parameter γ(0−1). Here it is seen that temperature ascends with expanding value of γ significantly in the boundary region 0.4≤ξ≤2.5 (approximately). Additionally, the Figure 4c illustrates the temperature distribution against Stefan blowing parameter Sb. A critical deviations in profile of temperature can be seen in this figure. With increase in the value of Sb, velocity increases as depicted in Figure 4c. The slow impact of initial volume fraction $\phi_{1}$ over the temperature profile can be depicted in Figure 4d.

Figure 4e shows the impact of Brownian movement boundary Nb (0.001 to 0.05) over nanoparticle temperature appropriation. At the point when fluid molecules hit with one another, it makes a subjective movement among themself called Brownian movement, which thusly increases the boundary layer thickness and subsequently nanoparticle temperature enhances for additional increment of Brownian movement Nb which further results in decrement of Nusselt number. Figure 4f shows the impact of nanoparticle temperature under the influence of thermophoresis Nt (0.001 to 0.05). With an expansion in the estimations of thermophoresis parameter Nt, the temperature inclination tumbles down.

### 4.2. Impact of Controlling Parameters on Nanoparticle Volume Fraction and Motile Density Microorganisms Distribution

**Figure 5:**
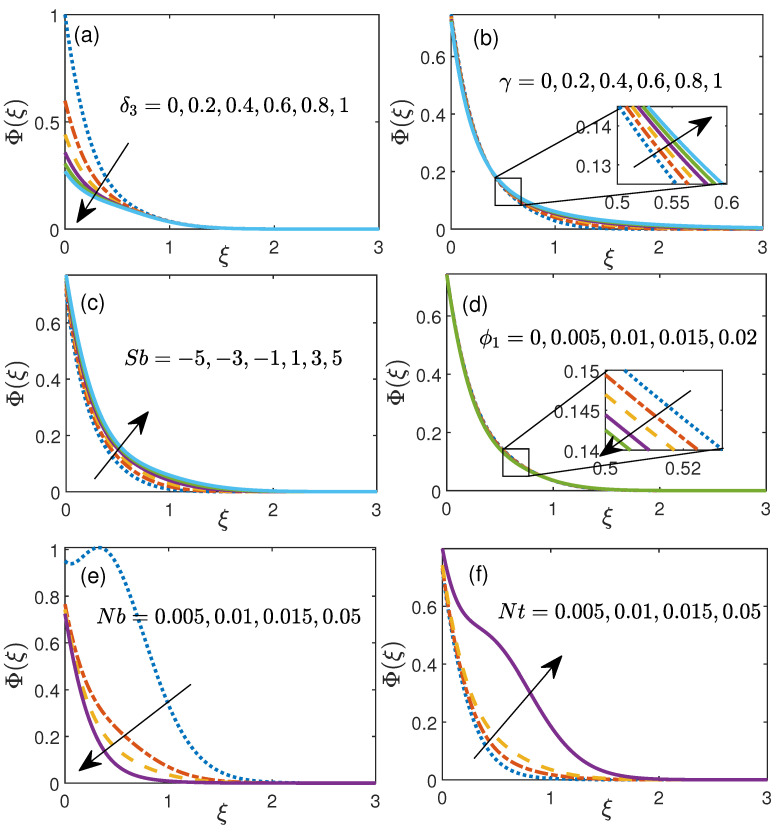
Volume fraction profile for (**a**) slip parameter $\delta_{3}$, (**b**) curvature parameter γ, (**c**) Stefan blowing Sb, (**d**) initial volume fraction $\phi_{1}$, (**e**) Brownian motion Nb, (**f**) thermophoresis Nt.

The nanoparticle volume fraction profile for six physical fluid parameters is illustrated via Figure 5. Figure 5a portrays the impact of the slip $\delta_{3}$ (0 to 1) on focus conveyance. As we continue expanding the estimation of slip parameter $\delta_{3}$, the liquid fixation diminishes because of the mass slip. The charts are particular inside 0.0≤ξ≤1.7 (roughly) and past that area, the end result is not huge. Moreover, the diagrams of fixation are heightened for lower estimations of the $\delta_{3}$. Figure 5b represents the nanoparticle volume fraction Φ(ξ) against curvature parameter γ in the range 0 to 1. With an augmentation in the value of curvature parameter γ, volume fraction rises as shown in the plot Figure 5b. As the curvature parameter (γ) becomes zero, the present problem is converted to flat stretching sheet problem which justifies the decrement in boundary layer.

Furthermore, Figure 5c manifests the effect of Stefan blowing parameter Sb (−5 to 5) on nanoparticle volume fraction. Here, this graph shows that, volume fraction enhances with an increment in Sb. On the other hand, nanoparticle volume fraction declines for higher values of $\phi_{1}$ (0 to 0.02) as depicted in Figure 5d. Figure 5e shows the effect of Nb (0.001 to 0.05) on profile of nanoparticle volume fraction. With an increment in Nb, nanoparticles slam into one another with higher speed which brings about diminishing of nanoparticle volume fraction and subsequently, the Sherwood number lessens as portrayed in Table 5. Figure 5f representations variety of nanoparticle volume fraction against Nt. This diagram shows that with an increment in thermophoresis Nt, nanoparticle volume fraction upgrades. Fundamentally, in the event of thermophoresis applied by a molecule on the other molecule will produce the development of particle movement from more sizzling to colder part and thus strengthening in the nanoparticle volume fraction is noticed through Figure 5f.

**Figure 6:**
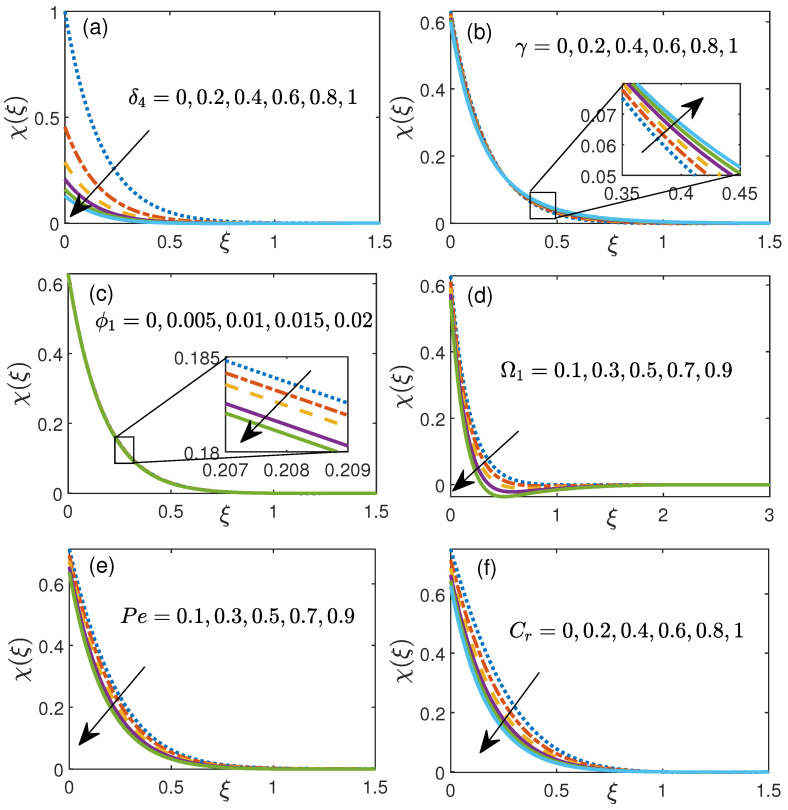
Motile density micro-organism distribution for (**a**) motile slip parameter $\delta_{4}$, (**b**) curvature parameter γ, (**c**) initial volume fraction $\phi_{1}$, (**d**) concentration difference parameter $\Omega_{1}$, (**e**) Peclet number Pe, (**f**) chemical reaction $C_{r}$.

Figure 6a shows the influence of micro-organism slip parameter $\delta_{4}$ (0 to 1) over motile concentration. This figure elaborates that motile concentration falls with rise in motile slip parameter $\delta_{4}$. Figure 6b manifests the impact of curvature parameter γ (0–1) on χ(ξ). An enhancement in the value of motile concentration is noticed for higher value of curvature parameter γ. Additionally, motile concentration decreases for higher values of initial volume fraction $\phi_{1}$ and concentration difference parameter $\Omega_{1}$ as shown in Figure 6c,d respectively. It is imagined that the non-negative estimations of Peclet number Pe subverts the thickness of gyrotactic micro-organisms since more Pe improves the movement of liquid particles prompting more slender micro-organisms as seen in Figure 6e. Figure 6f illustrates motile concentration χ(ξ) against the chemical reaction parameter $C_{r}$ (0 to 1) and this shows that motile concentration falls with rise in chemical reaction parameter $C_{r}$.

### 4.3. Influence on Skin Friction Coefficient and Nusselt Number with Different Controlling Parameter

**Figure 7:**
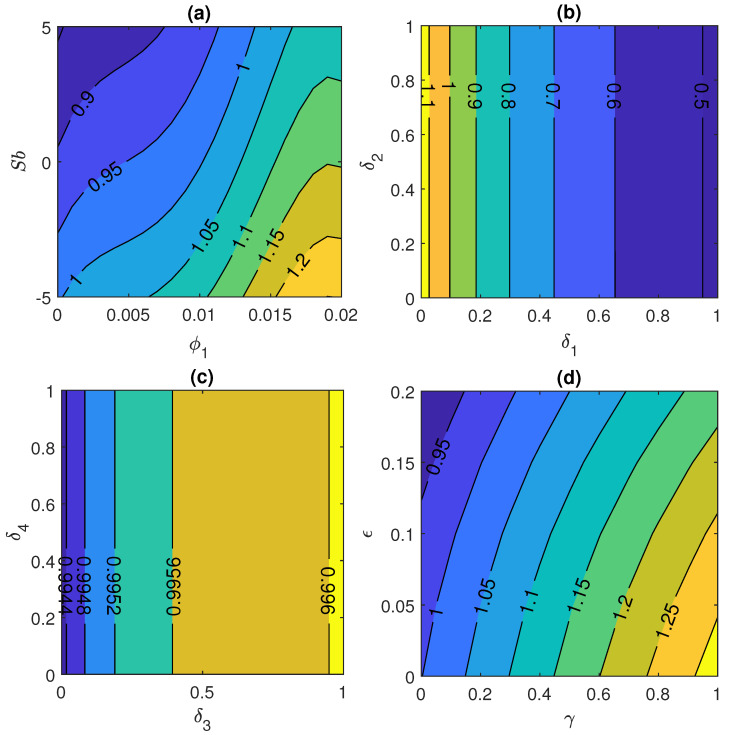
Skin friction coefficient for (**a**) Stefan blowing Sb and initial volume fraction $\phi_{1}$, (**b**) velocity slip parameter $\delta_{1}$ and thermal slip parameter $\delta_{2}$, (**c**) volume fraction slip parameter $\delta_{3}$ and micro-organism slip parameter $\delta_{4}$ and (**d**) free stream velocity ϵ and curvature parameter γ.

Figure 7a represents the variation of skin friction coefficient $Cf_{r}$ against Stefan blowing parameter Sb and initial volume fraction $\phi_{1}$ and this figure shows that skin friction coefficient enhances when the values of Sb and $\phi_{1}$ rises. Figure 7b shows the effect of skin friction coefficient under the influence of velocity slip parameter $\delta_{1}$ and thermal slip parameter $\delta_{2}$. This plot illustrates that skin friction declines with enhancement in $\delta_{1}$ and $\delta_{2}$. Moreover the effect of $\delta_{2}$ is not more prominent in this case as seen in Figure 7b. While reverse impact is noticed in case of Figure 7c and in this plot, slip parameter plays an important role to increase the value of skin friction coefficient ranging from $0.9944\leq Cf_{r}\leq 0.996$ when volume fraction slip parameter and motile concentration slip parameter are in the range from 0 to 1. Furthermore, Figure 7d manifests that $Cf_{r}$ rises with rise in free stream velocity parameter, when 0≤ϵ≤0.2 and curvature parameter, when 0≤γ≤1.

**Figure 8:**
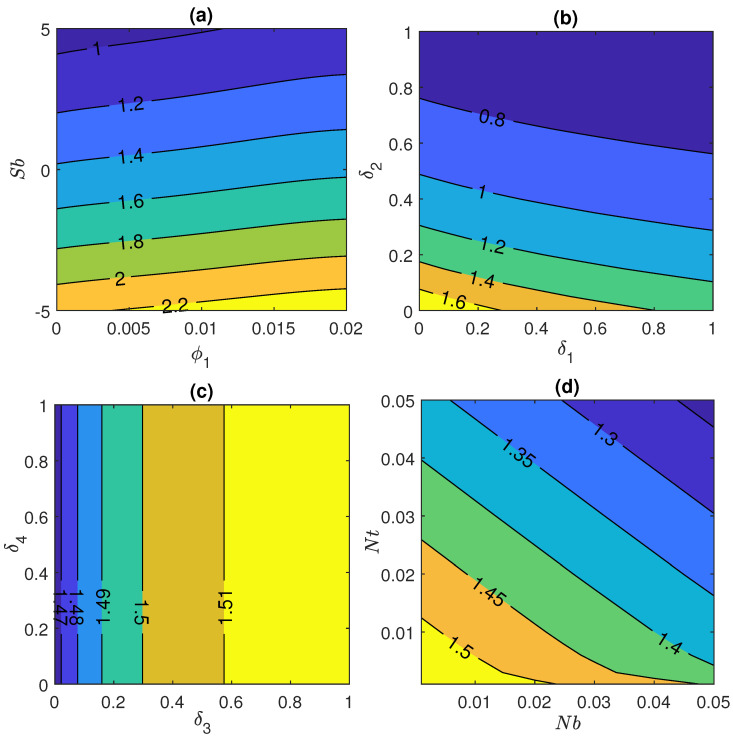
Nusselt number for (**a**) Stefan blowing Sb and initial volume fraction $\phi_{1}$, (**b**) velocity slip parameter $\delta_{1}$ and thermal slip parameter $\delta_{2}$, (**c**) volume fraction slip parameter $\delta_{3}$ and motile concentration slip $\delta_{4}$ and (**d**) Brownian motion Nb and thermophoresis Nt.

Combined effect of prominent fluid parameters Sb, $\phi_{1}$, multiple slips and Nt & Nb on heat transfer rate has been illustrated via Figure 8a–d respectively. Rate of heat transportation falls with rise in Stefan blowing parameter, when −5≤Sb≤5 and initial volume fraction $\phi_{1}$ ranging from 0 to 0.02. Additionally, this plot shows that the effect of Sb is more dominant in comparison to $\phi_{1}$ that can be seen in Figure 8a very clearly. Moreover same pattern is observed in case of Figure 8b when combined effect of $0\leq \delta_{1}\leq 1$ and $0\leq \delta_{2}\leq 1$ are taken into account.

Afterthat, Figure 8c describes the variation in heat transfer under the impact of $\delta_{4}$ and $\delta_{3}$. It is noted from the graph that heat transfer rate increases with only variation in volume fraction slip parameter as a negligible effect is found in case of motile concentration density parameter. Furthermore, the joined impact of higher Nt and Nb causes the rate of heat transfer to decline as shown in Figure 8d via contour plot.

### 4.4. Impact of Prominent Physical Parameters on Sherwood Number and Motile Microorganism Number

**Figure 9:**
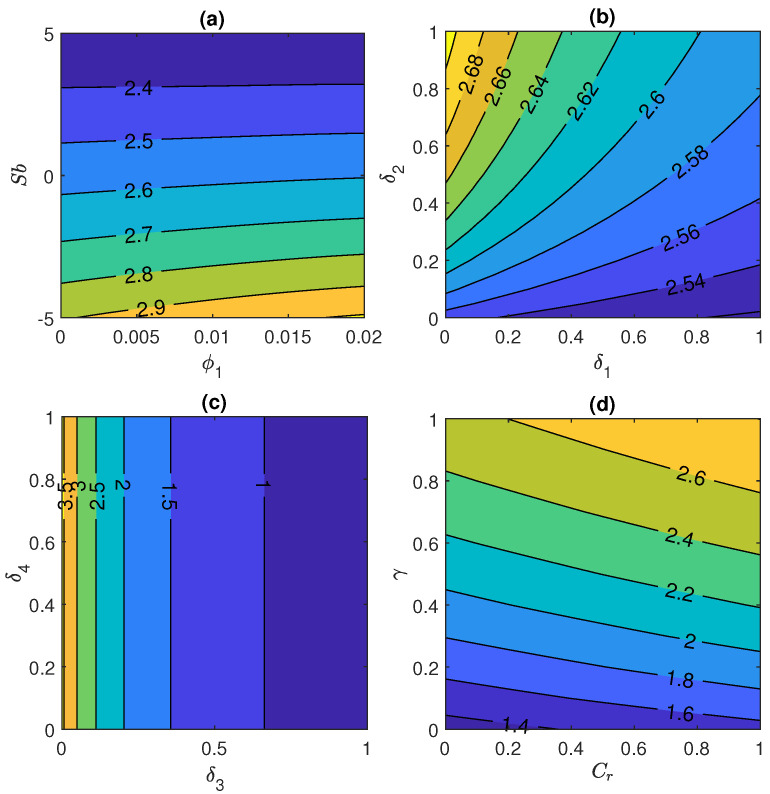
Sherwood number for (**a**) Stefan blowing Sb and initial volume fraction $\phi_{1}$, (**b**) velocity slip parameter $\delta_{1}$ and thermal slip parameter $\delta_{2}$, (**c**) volume fraction slip parameter $\delta_{3}$ and motile concentration slip parameter $\delta_{4}$ and (**d**) Chemical reaction $C_{r}$ and curvature parameter γ.

**Figure 10:**
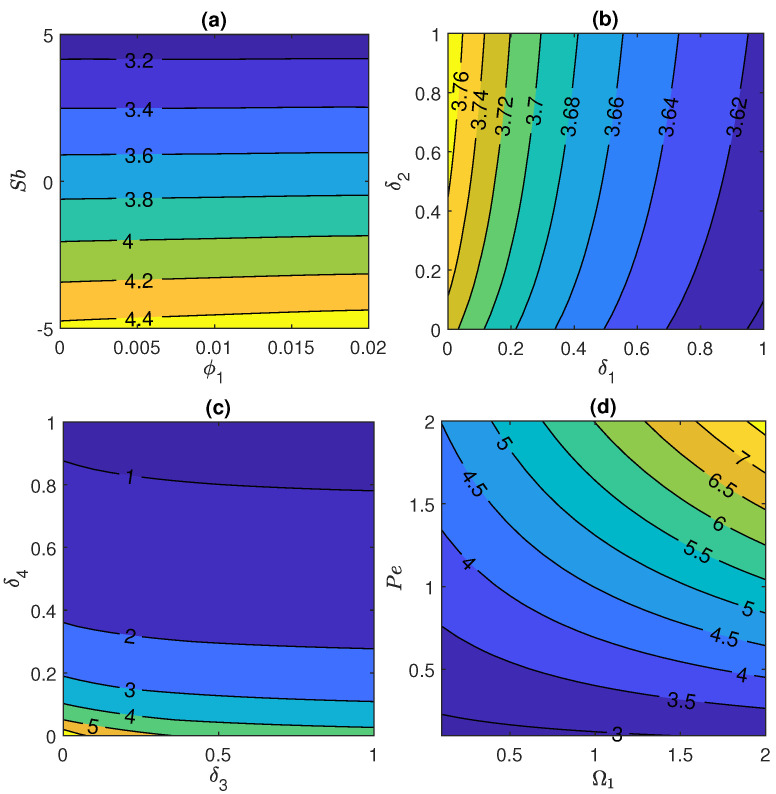
Motile micro-organism density number for (**a**) Stefan blowing Sb and initial volume fraction $\phi_{1}$, (**b**) velocity slip parameter $\delta_{1}$ and thermal slip parameter $\delta_{2}$, (**c**) volume fraction slip parameter $\delta_{3}$ and motile concentration slip parameter $\delta_{4}$ and (**d**) concentration difference parameter $\Omega_{1}$ and Peclet number Pe.

Figure 9 depicts the impact of various physical parameters on Sherwood number. The rate of mass transportation declines with the rise in −5≤Sb≤5 and $\phi_{1}$ both (as shown in Figure 9a) while it rises with the rise in $\delta_{2}$ and $\delta_{1}$ (as shown in Figure 9b). Additionally, rate of mass transportation declines when combined effect of higher $\delta_{3}$ and $\delta_{4}$ has been taken into account as can be seen in Figure 9c. Further, Figure 9d shows that Sherwood number enhances with enhancement in the values of $C_{r}$ and γ. Figure 10 represents the motile micro-organism number distribution under the influence of crucial fluid parameters. In this plot, Figure 10a shows that, mass transportation of micro-organisms decreases for higher values of Sb and $\phi_{1}$. Furthermore, same impact is noticed in case of higher $\delta_{1}$ and $\delta_{2}$ (see Figure 10b) and in case of higher $\delta_{3}$ and $\delta_{4}$ (see Figure 10c) while reverse impact is noticed for higher Pe and $\Omega_{1}$ as shown in Figure 10d.

## 5. Conclusions

The present study focused on numerical investigation of Stefan blowing MHD hybrid nanofluid flow induced by stretching cylinder by considering Cattaneo–Christov heat flux and mass flux using modified Buongiorno’s nanofluid model. The main fallout of the present study incorporating the significance on skin friction and heat transfer is as follows:

1. Stefan blowing and initial nanoparticle volume fraction are found to have maximum impact on skin friction. The optimal (minimum) value of skin friction is recorded for the lower value of initial nanoparticle volume fraction and higher value of Stefan blowing parameter, which is required to have better flow performance and to avoid abrasion.
2. The consideration of velocity slip has a detrimental effect on skin friction to nearly 50% for the unit increment in its value. However, the skin friction are independent of variation in other slip conditions (thermal, nanoparticle and micro-organism).
3. Higher values of the free stream velocity reduce the skin friction but the curvature parameter has a contrary impact on it.
4. Heat transfer enhancement of upto 20% is noticed with increment of 2% of initial volume fraction $\phi_{1}$ of hybrid nanomaterials. With controlled nanoparticle volume fraction, the heat transfer can be optimized required for several industrial processings. Additionally, velocity and thermal slips have considerable impact on the Nusselt number.
5. The nanoparticle volume fraction upsurges with an extended zigzag motion of nanoparticles and the declined thermo-migration of nanoparticles.
6. The curvature parameter and chemically reactive nanoparticles both favor the mass transfer. Even the Sherwood number gets a boost with the increment in initial nanoparticle volume fraction.
7. An excellent agreement is noticed between the numerical results obtained from the Finite Element Method and MATLAB *bvp5c* routine.

The current biological convection model with the involvement of hybrid nanoparticles has many applications in interdisciplinary scientific fields such as biomedicine, biofuel biotechnology, heat exchangers, and enzyme-based biosensors. Our future research will examine rheological properties, especially their impact on microbial transmission, to assess the upcoming announced potential characteristics of new biofuel cells and other technological applications.

## Figures and Tables

**Table 1:** Thermophysical properties of basefluid and nanoparticles (Ag and MgO).

	Ag	MgO	Pure Water
ρ (Kg/m${}^{3}$)	10,500	3560	997.1
*c* (J/Kg K)	235	955	4179
*k* (W/m K)	429	45	0.62
σ (S/m)	6.21× $10^{7}$	≈$10^{-8}$	0.05
μ (Kg/m S)	-	-	8.55 × $10^{-4}$

**Table 2:** Mesh independency test with linear shape function (m=2) and boundary layer thickness ($\xi_{\infty }$).

**Element Size**	$\xi_{\infty }=6$				$\xi_{\infty }=8$			
	${\mathrm{Cf}}_{r}$	${\mathrm{Nu}}_{r}$	${\mathrm{Sh}}_{r}$	$M_{r}$	${\mathrm{Cf}}_{r}$	${\mathrm{Nu}}_{r}$	${\mathrm{Sh}}_{r}$	$M_{r}$
0.1	0.995848	1.482866	2.584126	3.731891	0.995449	1.483016	2.584130	3.731912
0.05	0.995469	1.483159	2.576088	3.716691	0.995070	1.483313	2.576094	3.716716
0.025	0.995375	1.483235	2.574064	3.712841	0.994975	1.483390	2.574071	3.712867
0.01	0.995348	1.483256	2.573496	3.711759	0.994949	1.483412	2.573503	3.711785
0.005	0.995344	1.483259	2.573415	3.711604	0.994945	1.483415	2.573422	3.711630
**Element Size**	$\xi_{\infty }=10$				$\xi_{\infty }=12$			
	${\mathrm{Cf}}_{r}$	${\mathrm{Nu}}_{r}$	${\mathrm{Sh}}_{r}$	$M_{r}$	${\mathrm{Cf}}_{r}$	${\mathrm{Nu}}_{r}$	${\mathrm{Sh}}_{r}$	$M_{r}$
0.1	0.995390	1.483037	2.584131	3.731914	0.995380	1.483041	2.584131	3.731915
0.05	0.995011	1.483335	2.576095	3.716720	0.995002	1.483339	2.576095	3.716720
0.025	0.994916	1.483412	2.574072	3.712870	0.994907	1.483416	2.574072	3.712871
0.01	0.994890	1.483434	2.573504	3.711789	0.994880	1.483437	2.573504	3.711789
0.005	0.994886	1.483437	2.573423	3.711634	0.994876	1.483440	2.573423	3.711635

**Table 3:** Mesh independency test with quadratic shape function (m=3) and boundary layer thickness ($\xi_{\infty }$).

**Element Size**	$\xi_{\infty }=6$				$\xi_{\infty }=8$			
	${\mathrm{Cf}}_{r}$	${\mathrm{Nu}}_{r}$	${\mathrm{Sh}}_{r}$	$M_{r}$	${\mathrm{Cf}}_{r}$	${\mathrm{Nu}}_{r}$	${\mathrm{Sh}}_{r}$	$M_{r}$
0.1	0.995288	1.482829	2.573205	3.700777	0.994889	1.482984	2.573212	3.700801
0.05	0.995329	1.483153	2.573335	3.704312	0.994930	1.483308	2.573342	3.704338
0.025	0.995340	1.483233	2.573374	3.707449	0.994940	1.483389	2.573381	3.707475
0.01	0.995342	1.483256	2.573386	3.709793	0.994943	1.483412	2.573393	3.709819
0.005	0.995343	1.483259	2.573387	3.710653	0.994944	1.483415	2.573394	3.710679
**Element Size**	$\xi_{\infty }=10$				$\xi_{\infty }=12$			
	${\mathrm{Cf}}_{r}$	${\mathrm{Nu}}_{r}$	${\mathrm{Sh}}_{r}$	$M_{r}$	${\mathrm{Cf}}_{r}$	${\mathrm{Nu}}_{r}$	${\mathrm{Sh}}_{r}$	$M_{r}$
0.1	0.994830	1.483005	2.573213	3.700804	0.994820	1.483009	2.573213	3.700805
0.05	0.994871	1.483330	2.573343	3.704341	0.994861	1.483334	2.573344	3.704342
0.025	0.994881	1.483411	2.573382	3.707479	0.994872	1.483415	2.573383	3.707480
0.01	0.994884	1.483434	2.573394	3.709823	0.994875	1.483437	2.573394	3.709824
0.005	0.994885	1.483437	2.573395	3.710683	0.994875	1.483440	2.573396	3.710684

**Table 4:** Validation of present FEM results with MATLAB *bvp5c* for the parametric values; $\alpha_{t}=\alpha_{c}=M=\epsilon =\beta_{i}\left(1,\ldots ,4\right)=\Omega_{1}=\gamma =0.1$, Nb=Nt=0.01, Pr=6.2, Le=Lb=5, $C_{r}=Pe=1$.

Sb	$\phi_{1}$	FEM		MATLAB *bvp5c*	
		$-f^{\prime \prime }\left(0\right)$	$-\theta^{\prime }\left(0\right)$	$-f^{\prime \prime }\left(0\right)$	$-\theta^{\prime }\left(0\right)$
−2	0.01	0.92401060	1.71063232	0.92401055	1.71063225
−1		0.90400142	1.54416075	0.90400137	1.54416073
0		0.88413285	1.38255544	0.88413281	1.38255547
1		0.86454938	1.22697477	0.86454934	1.22697487
2		0.84541153	1.07879608	0.84541149	1.07879626
1	0	0.88744497	1.26305925	0.88744493	1.26305936
	0.005	0.87633789	1.24418922	0.87633785	1.24418932
	0.015	0.82487969	1.21973533	0.82487965	1.21973542
	0.02	0.81263218	1.20750268	0.81263215	1.20750276

**Table 5:** Variations in quantities of interest with magnetic field, Stefan blowing, curvature, free stream and initial nanoparticle volume fraction parameters.

*M*	Sb	γ	ϵ	$\phi_{1}$	${\mathrm{Cf}}_{r}$	${\mathrm{Nu}}_{r}$	${\mathrm{Sh}}_{r}$	$M_{r}$
0	0.1	0.1	0.1	0.01	0.958815	1.492518	2.574126	3.724582
0.2					1.029288	1.474745	2.572737	3.720466
0.5					1.124164	1.450629	2.571015	3.714981
1					1.261306	1.415459	2.568802	3.707166
	−3				1.048829	1.910737	2.785927	4.166730
	−1				1.012783	1.622628	2.643372	3.874408
	0				0.996462	1.495597	2.579568	3.736039
	1				0.981118	1.378151	2.519442	3.602502
	3				0.953033	1.168544	2.407608	3.349496
		0			0.961862	1.460638	2.545320	3.689000
		0.2			1.027009	1.506224	2.600866	3.755347
		0.5			1.118951	1.574069	2.679831	3.850559
		1			1.261265	1.683889	2.801731	3.998840
			0		1.033387	1.469748	2.572731	3.720044
			0.05		1.016872	1.475782	2.573006	3.721086
			0.1		0.994885	1.483438	2.573403	3.722471
			0.2		0.936694	1.502411	2.574580	3.726132
				0	0.906476	1.412154	2.556206	3.715626
				0.002	0.932973	1.428518	2.559989	3.717488
				0.01	0.994885	1.483438	2.573403	3.722471
				0.02	1.144794	1.553461	2.587661	3.730159

**Table 6:** Variation in quantities of interest with slip parameters, chemical reaction and nanofluid parameters.

$\delta_{1}$	$\delta_{2}$	$\delta_{3}$	$\delta_{4}$	$C_{r}$	Nt	Nb	${\mathrm{Cf}}_{r}$	${\mathrm{Nu}}_{r}$	${\mathrm{Sh}}_{r}$	$M_{r}$
0	0.1	0.1	0.1	1	0.01	0.01	1.142372	1.545323	2.584920	3.749584
0.2							0.884582	1.432896	2.564600	3.700998
0.5							0.671345	1.321554	2.546924	3.655857
1							0.486269	1.203998	2.530404	3.611454
	0						0.994904	1.712409	2.543340	3.713681
	0.2						0.994871	1.307643	2.596794	3.729352
	0.5						0.994843	0.963149	2.643405	3.743163
	1						0.994818	0.668176	2.684117	3.755327
		0					0.994276	1.465529	3.568595	4.044178
		0.2					0.995230	1.493596	2.011039	3.531668
		0.5					0.995718	1.508042	1.213800	3.249761
		1					0.996014	1.516824	0.730580	3.072294
			0				0.994885	1.483438	2.573403	5.845095
			0.2				0.994885	1.483438	2.573403	2.730793
			0.5				0.994885	1.483438	2.573403	1.517774
			1				0.994885	1.483438	2.573403	0.872117
				0			0.995640	1.492384	1.341482	2.486181
				0.2			0.995427	1.489573	1.689495	2.852012
				0.5			0.995181	1.486593	2.090375	3.255614
				1			0.994885	1.483438	2.573403	3.722471
					0.001		0.994773	1.509164	2.757261	3.777386
					0.005		0.994825	1.497691	2.672910	3.751970
					0.01		0.994885	1.483438	2.573403	3.722471
					0.05		0.995236	1.373002	2.000487	3.575400
						0.001	0.996163	1.527152	0.488376	3.054297
						0.005	0.995024	1.503198	2.346370	3.651667
						0.01	0.994885	1.483438	2.573403	3.722471
						0.05	0.994775	1.338567	2.753463	3.778114
